# Safety and Effectiveness of Concomitant Mitral Transcatheter Edge-to-Edge Repair and Left Atrial Appendage Closure

**DOI:** 10.3390/jcm12144742

**Published:** 2023-07-18

**Authors:** Marco Frazzetto, Claudio Sanfilippo, Giuliano Costa, Salvatore Scandura, Giuseppe Castania, Jessica De Santis, Maria Sanfilippo, Maria Elena Di Salvo, Salvatore Uccello, Gerardo Rugiano, Sofia Rizzo, Chiara Barbera, Corrado Tamburino, Marco Barbanti, Carmelo Grasso

**Affiliations:** 1 Division of Cardiology, A.O.U. Policlinico “G. Rodolico San Marco”, 95123 Catania, Italy; marcofrazzetto7@gmail.com (M.F.); cla.sanfi24@gmail.com (C.S.); giulianocosta90@gmail.com (G.C.); salvatore.scandura@tin.it (S.S.); giuseppe.castania16@gmail.com (G.C.); jessicadesantis3@gmail.com (J.D.S.); sanfilippomariap@gmail.com (M.S.); mari.disalvo@gmail.com (M.E.D.S.); uccellosalvo@gmail.com (S.U.); gerardorugiano@gmail.com (G.R.); sofiariz@hotmail.it (S.R.); chiara.barbera.me@gmail.com (C.B.); tambucor@unict.it (C.T.); melfat75@gmail.com (C.G.); 2 Faculty of Medicine and Surgery, Università degli Studi di Enna “Kore”, 94100 Enna, Italy

**Keywords:** mitral transcatheter edge-to-edge repair, left atrial appendage closure, atrial fibrillation, mitral regurgitation, concomitant procedure

## Abstract

Background: Concomitant mitral transcatheter edge-to-edge repair (M-TEER) and left atrial appendage closure (LAAC) showed to be a feasible approach to optimize the treatment of patients eligible for both procedures, but mid-term outcomes are unclear. Methods: We retrospectively analyzed consecutive patients undergoing M-TEER and enrolled in the local prospective Getting Reduction of Mitral Insufficiency by Percutaneous Clip Implantation (GRASP) registry. We compared patients undergoing isolated M-TEER (*n* = 58, 58.5%) with those undergoing concomitant M-TEER and LAAC (*n* = 41, 41.5%) from January 2018 to December 2022. The primary endpoint was a composite of all-cause death, stroke or systemic embolism, hospitalization for heart failure, and bleeding at 1 year. The co-primary endpoint was procedural success. Results: The primary endpoint was similar between patients undergoing concomitant M-TEER+LAAC or isolated M-TEER (Kaplan Meier (KM) estimates 36.6% vs. 44.8%; p_log-rank_ = 0.75). Procedural success was also similar (92.7% vs. 94.8%; *p* = 0.69). At 1- year, minor bleeds were lower in patients undergoing concomitant M-TEER and LAAC (KM estimates 0.0% vs. 18.9%; p_log-rank_ < 0.01). Conclusion: In patients with concomitant MR and AF and eligible for M-TEER and LAAC treatment, a combined approach of M-TEER and LAAC was as safe as an M-TEER-alone strategy and associated with lower minor bleeding at 1 year.

## 1. Introduction

Atrial fibrillation (AF) is a frequent comorbidity in patients suffering from mitral regurgitation (MR). Subjects with MR are more likely to be affected by AF, largely imputable to atrial dilatation and electrophysiological remodeling frequently caused by regurgitant mitral flow. The presence of these arrhythmias adds different challenges in the management and treatment of patients with MR. Indeed, it often amplifies patients’ symptoms due to the paroxysm of the disturbance or the high ventricular response rate, and requires an anticoagulation therapy to avoid thromboembolic events in a population that often has a higher bleeding risk per se. As a corollary, managing these patients requires a comprehensive approach that addresses both MR and AF.

In 1996, Blackshear et al. first highlighted that the left atrial appendage was the primary source of thromboembolism in non-valvular AF (91%), and afterwards different studies established the role of left atrial appendage closure (LAAC) in preventing thromboembolic strokes [1,2]. 

Mitral transcatheter edge-to-edge repair (M-TEER) is currently the therapy of choice for patients affected by severe MR not eligible for surgery [3]. 

In randomized clinical trials on M-TEER, AF is frequently observed in a significant proportion of patients, ranging from 33% to 57% of cases [4,5,6].

Most of these patients had a high bleeding risk or had contraindications for oral anticoagulation. In this setting, transcatheter LAAC has shown its safety and efficacy for both reducing hemorrhagic events and cardioembolic strokes [7]. 

Over the past two decades, several transcatheter techniques and devices have been developed for the transcatheter treatment of mitral regurgitation and LAAC.

LAAC can be performed as a staged procedure or concomitant with M-TEER.

Combining M-TEER and LAAC seems an attractive strategy, avoiding repeat left atrial access in two different procedures. However, data about safety and effectiveness of this concomitant approach are scarce [8,9]. 

In this study, we aimed at reporting safety and mid-term outcomes of concomitant M-TEER and LAAC procedures, comparing them to those of patients undergoing isolated M-TEER throughout the same period.

This study has the potential to give valuable insights into the potential benefits and risks associated with performing M-TEER and LAAC in the same setting, contributing to generate evidence that may guide clinicians towards the optimization of these patients’ managements.

## 2. Materials and Methods

**Figure 1:**
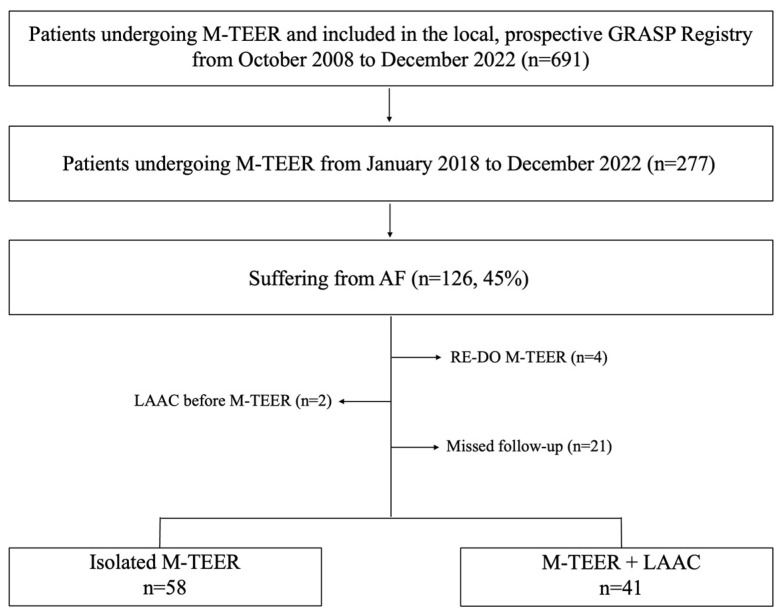
Study flow chart. AF, atrial fibrillation; M-TEER, mitral transcatheter edge-to-edge repair; LAAC, left atrial appendage closure.

From January 2018 to December 2022 a total of 126 consecutive patients with severe MR affected by AF underwent isolated M-TEER or concomitant M-TEER+LAAC at our institution as part of the ongoing, local prospective Getting Reduction of Mitral Insufficiency by Percutaneous Clip Implantation (GRASP) registry. The study was conducted in accordance with the Declaration of Helsinki and details of the registry have been previously described [10]. Forty-one patients (41.5%) undergoing concomitant M-TEER and LAAC were compared to 58 patients (58.5%) undergoing isolated M-TEER. Exclusion criteria were (1) lack of follow-up, (2) repeated M-TEER procedures, and (3) LAAC procedures performed before M-TEER procedures (Figure 1).

Indications for isolated M-TEER or concomitant M-TEER+LAAC were decided by the local heart team according to current guidelines.

Mitral regurgitation was graded according to the current guidelines of the European Society of Cardiology [3].

M-TEER was performed using a 24 Fr MitraClip (Abbott Vascular, Santa Clara, CA, USA) or 22 Fr Pascal system (Edwards Lifescience, Irvine, CA, USA) under general anesthesia with transesophageal echocardiographic (TOE) and fluoroscopic guidance. Patients received either third- (NTR and XTR) or fourth- (NT, NTW, XT, XTW) generation MitraClip or Pascal (P10 and Ace) devices.

The right femoral vein was used as the access of choice for the procedures. Initially, a 7-F introducer was inserted, and then exchanged with an 8-F Mullins sheath (Abbott Vascular, Minneapolis, MN) over a 0.32 guidewire. The transseptal puncture was performed using a Brockenbrough needle under TOE guidance.

Invasive arterial pressure was monitored during the procedures through the left femoral artery, and a central venous catheter was placed in the left femoral vein for fluid and drug infusions.

Patients without a coronary angiogram within 1 year of the procedure underwent repeat coronary angiography during the index TEER procedure.

The LAAC procedure was performed systematically after M-TEER, through the same transseptal puncture site using the 14Fr TrueSeal Access System for the Watchman FLX device or the 14 Fr TorqVue delivery sheath for the Amulet device. The type and size of the device for LAAC procedure was decided intraoperatively according to TOE findings. After withdrawal of M-TEER system, the LAAC system was inserted and the ProGlide (Abbott Vascular, Santa Clara, CA, USA) or the figure of 8 stitch was partially tightened over it to ensure hemostasis for the rest of the procedure. At 45 days, TOE was systematically performed to exclude residual peri-device leak and device-related thrombosis. 

Clinical follow-up data were obtained by telephone interviews and review of medical records. 

### 2.1. Study Endpoints

The primary endpoint was the composite of all-cause death, stroke or systemic embolism, hospitalization for heart failure (HF), and bleedings at 1 year. 

The co-primary endpoint was procedural success. In the case of concomitant M-TEER and LAAC groups, procedural success was defined when both procedures were successful.

Secondary endpoints were each single component of the primary endpoint, technical success, and vascular complications (other than local hematoma). 

All endpoints were defined according to the Mitral Valve Academic Research Consortium classification (MVARC), the Munich Consensus, and the Bleeding Academic Research Consortium classification (BARC) [11,12,13]. 

### 2.2. Statistical Analysis

Continuous variables are presented as median and interquartile range (IQR); categorical variables are presented as number and percentage. The Kolmogorov–Smirnov test was used to test for a normal distribution of continuous variables. The two groups were compared using the Mann–Whitney U test for continuous variables and the chi-square test for categorical variables. Time-to-first-event rates for the study endpoints were estimated using the Kaplan–Meier method. 

Multivariable logistic regression analysis was performed to assess baseline factors associated with the primary composite endpoint. The results were reported as odds ratio (OR) with a 95% confidence interval (CI). All statistical tests were performed 2-tailed, and a *p*-value < 0.05 was considered the threshold for statistical significance. All statistical analyses were performed using SPSS version 27 (IBM software).

## 3. Results

### 3.1. Population

Baseline characteristics of the study population are presented in the Table 1. 

The median age was 79 years (IQR: 73–82) and 45% of patients were female. Patients had diabetes and hypertension in 32.3% and 83.8% of cases, respectively. 

The estimated mortality risks according to Society of Thoracic Surgeons (STS) score and EUROScore II were similar between patients undergoing concomitant M-TEER+LAAC or isolated M-TEER (STS score 7.7% vs. 5.3%; EUROScore II 8.0% vs. 5.6%; *p* = 0.22 and *p* = 0.08 respectively). Chronic kidney disease (CKD) was more frequent in patients undergoing concomitant M-TEER+LAAC (73.1% vs. 53.4%; *p* = 0.04). 

Permanent, persistent, and paroxysmal AF were reported in 63%, 17%, and 20% of patients undergoing M-TEER+LAAC, respectively, and in 67%, 21%, and 12% of patients undergoing isolated M-TEER, respectively. 

The median CHA2DS2-VASc score and HAS-BLED score were 4 (IQR: 3–5) and 3 (IQR: 2–4), respectively, being higher in patients undergoing concomitant M-TEER+LAAC (median 5 vs. 4, *p* = 0.05) and (median 3 vs. 2.5 *p* < 0.01), respectively. 

The etiology of mitral regurgitation was similar between the concomitant and isolated procedure groups. Considering the entire population, 23% had degenerative mitral regurgitation, 67% had functional mitral regurgitation, and 10% had mixed mitral regurgitation (*p* = 0.95, *p* = 0.91 and *p* = 0.92, respectively). 

Chicken wing was the predominant morphology of left atrial appendage (54% of the cases), followed by cauliflower and windsock (17% for both).

### 3.2. Procedural Characteristics

Procedural characteristics are reported in Table 2.

The concomitant M-TEER+LAAC group received a greater amount of contrast than the isolated M-TEER group (100 (IQR 67–160) vs. 30 [IQR 20–50] mL; *p* < 0.01). 

Procedural time was similar between the combined and the isolated groups (median 75 (IQR 63–81) vs. 71 (IQR 61–78) min; *p* = 0.24).

Median fluoroscopy time was similar between the two groups (median 22 (IQR 14–28) vs. 21 (IQR 12–32) min, for patients undergoing M-TEER+LAAC and isolated TEER respectively; *p* = 0.64). 

The MitraClip was the most used device for M-TEER (97.9%) and a single clip was mainly used for the procedure.

The most used device for LAAC was the Watchman FLX (97.5%), and the most used access sheath was the double curve (85.4%).

The isolated M-TEER group underwent TOE evaluation at 45 days. One device related thrombus was found. No device embolization, leaks, or pericardial effusion were detected.

### 3.3. In-Hospital Outcomes

In-hospital outcomes are reported in Table 3.

No death, cardiac perforation, device embolization, acute kidney injury (AKI), or stroke occurred in either group. 

One major vascular complication occurred in a patient undergoing M-TEER+LAAC (2.4% vs. 0.0%; *p* = 0.41). It was due to pseudoaneurysm and successfully treated with surgical repair within the index hospitalization.

Seven minor vascular complications (5 local hematomas and 2 arteriovenous fistulas) occurred in patients undergoing the combined procedure, whereas 5 (4 local hematomas and 1 arteriovenous fistulas) occurred in those undergoing isolated M-TEER (17% vs. 8.6%; *p* = 0.20). All arteriovenous fistulas were successfully treated with femoral balloon angioplasty during the index procedure. 

**Figure 2:**
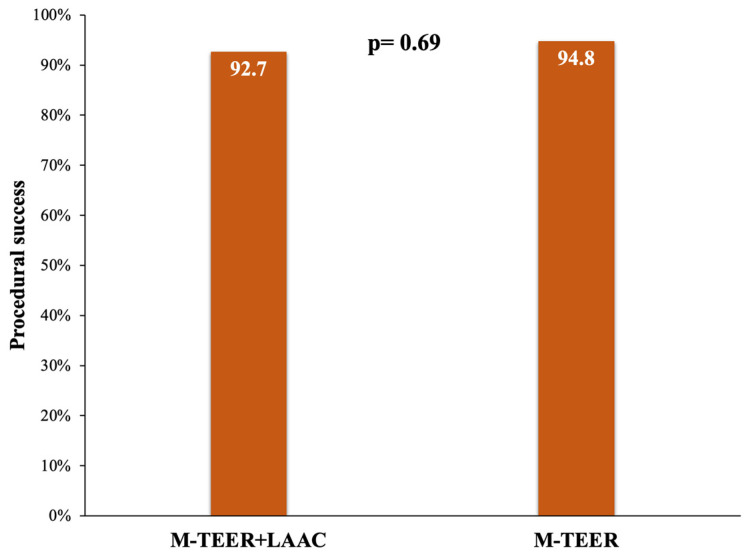
Primary endpoint. M-TEER, mitral transcatheter edge-to-edge repair; LAAC, left atrial appendage closure.

Procedural success was achieved in 94.8% and 92.7% of patients in the concomitant and isolated groups, respectively (*p* = 0.69) (Figure 2). 

A total of 6 patients did not achieve procedural success, 3 in each group. In the concomitant group, one case was due to an elevated mean gradient after leaflets grasping, one due to a left atrial appendage ostium exceeding the instruction for use of LAAC devices, and one due to a major vascular complication. In the isolated group, one was due to an elevated mean gradient after leaflets grasping, one due to clip detachment, and one due to the impossibility of guiding the procedure with bad TOE windows. 

**Figure 3:**
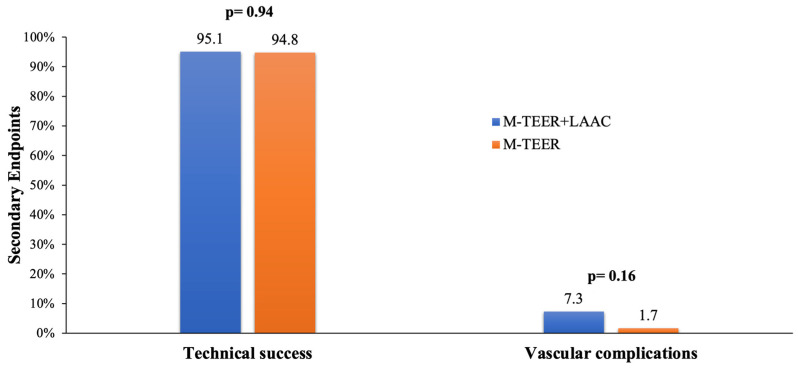
Secondary endpoints.

Technical success was achieved in 95.1% and 94.8% of patients in the concomitant and isolated groups, respectively (*p* = 0.94) (Figure 3). 

Procedure-related vascular complications did not differ between compared groups (concomitant 7.3% vs. isolated 1.7%, *p* = 0.16) (Figure 3).

Median length of hospital stay was 5 days in both groups (*p* = 0.36).

### 3.4. One-Year Outcomes

**Figure 4:**
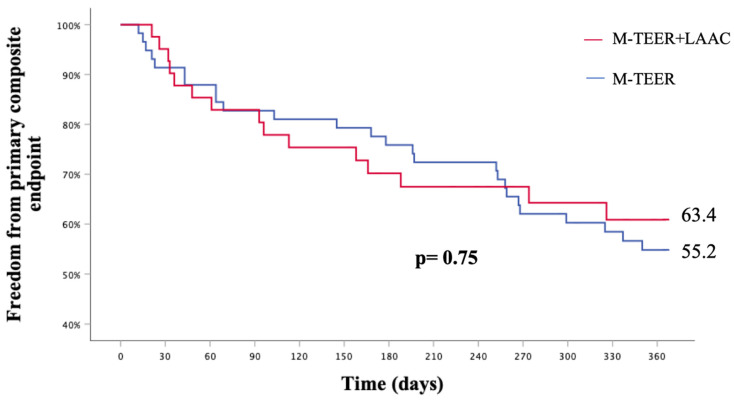
Kaplan–Meier estimate of freedom from the primary composite endpoint.

The composite primary endpoint did not differ between patients undergoing concomitant M-TEER+LAAC or isolated M-TEER (KM est. 33.6% vs. 44.8%, p_log-rank_ = 0.75) (Figure 4).

**Figure 5:**
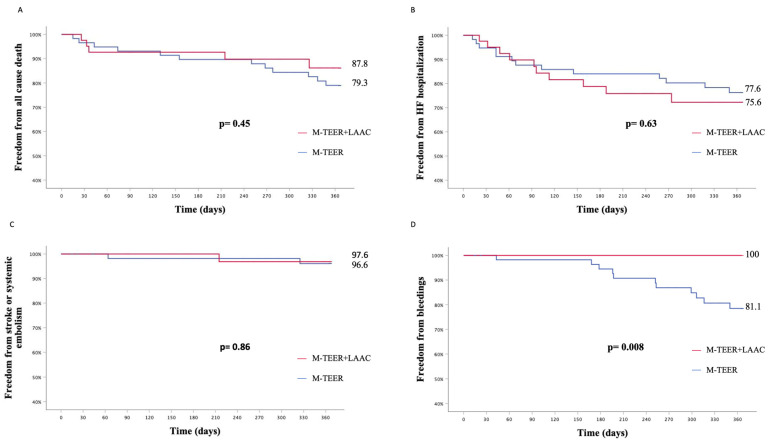
Secondary endpoints. Kaplan–Meier estimates for freedom from all-cause death (**A**), hospitalization for heart failure (**B**), stroke, or systemic embolism (**C**) and bleedings (**D**).

Taken separately, all-cause death (KM est. 12.2% vs. 20.7%, p_log-rank_ = 0.45), hospitalization for HF (KM est. 24.4% vs. 22.4%, p_log-rank_ = 0.63), stroke, or systemic embolism (KM est. 2.4% vs. 3.4%, p_log-rank_ = 0.86) rates did not differ between patients undergoing concomitant M-TEER+LAAC or isolated M-TEER (Figure 5).

A higher rate of bleeding events was reported in patients undergoing an isolated M-TEER procedure (KM est. 18.9% vs. 0.0%, p_log-rank_ = 0.008) (Figure 5). All bleeding events were adjudicated as minor bleeds (BARC 1 or 2): four epistaxis, four gastrointestinal hemorrhages, two hematuria, and one hemoptysis.

Multivariable logistic regression analyses of baseline characteristics associated with the primary composite endpoint are reported in Table 4. 

No predictors of the primary composite endpoint have been detected. 

## 4. Discussion

The prevalence of atrial fibrillation in patients undergoing M-TEER is high, and a not negligible portion of patients are at high bleeding risk or have contraindications for oral anticoagulation therapy [4,5,6,7,8,9,10,11,12,13,14,15]. During the past decade, LAAC showed to be an effective and attractive option against systemic cardio-embolism for patients that do not tolerate oral anticoagulation [16,17]. Notably, M-TEER and LAAC procedures share some key steps, such as venous access and left atrial catheterization. Recently, the combination of the two treatments into a single procedure showed to be safe and therefore seems an attractive solution for optimizing the treatment of this subset of patients. To date, few, small studies have shown the safety and effectiveness of the concomitant procedure [8,9]. 

We aimed at reporting the largest experience of performing concomitant M-TEER and LAAC procedures so far, comparing early and mid-term outcomes of consecutive patients included in our local prospective GRASP registry who underwent concomitant M-TEER+LAAC or isolated M-TEER procedures.

The main findings of this analysis were the following. 

First, the procedural and technical successes were high and comparable in the two groups. In a single case, it was not feasible performing LAAC, due to the large dimension of left atrial appendage ostium. Remarkably, in this study, the LAAC procedure was not planned with TOE or computed tomography angiography before the index procedure. One could assume that the concomitant approach may result in a lower success rate of LAAC, in consideration of a non-ideal transseptal puncture site. We showed that effective LAAC can be achieved even with a superior transeptal puncture and no prolongation of operative times, as suggested by the similar procedural and fluoroscopy time between the two study groups. 

Moreover, the current study was the first to assess the effectiveness of the use of the Watchman FLX device (95.1% of the cases) in consecutive patients undergoing concomitant M-TEER and LAAC procedures. It might be argued that the improvements brought by this device iteration have been determinant to the high procedural success, even using a suboptimal transeptal puncture site for LAAC.

The safety profile of the concomitant M-TEER+LAAC approach was supported by the absence of in-hospital adverse events (death, stroke, AKI, device embolization, or cardiac tamponade), apart from a single major vascular accident.

Of note, despite the concomitant group having more incidence of chronic kidney disease at baseline and receiving a higher volume of contrast medium, it did not result in higher AKI rates. 

Second, concomitant M-TEER+LAAC and isolated M-TEER had similar rates of all-cause death, HF rehospitalization, stroke, or systemic embolism at 1 year, but bleeding events were significantly lower in patients receiving LAAC (KM est. 18.9% vs. 0.0%, p_log-rank_ = 0.008). 

These results underline the effectiveness of adding LAAC during the index M-TEER procedure in eligible patients. Indeed, the possibility to withdraw oral anticoagulants after LAAC achieves the reduction of bleeding events in high bleeding risk patients without increasing the risk of ischemic events. In our study, only 9.7% of the patients who underwent LAAC continued with anticoagulation therapy at discharge, compared with 86.2% of subjects in the isolated M-TEER group.

Staging the two procedures is typically better reimbursed in most countries, but combining the two treatments into a single procedure avoids patients undergoing repeat vascular access and transseptal puncture, thus lowering the procedural risk of a two-stage approach. Although the study should be considered hypothesis-generating due to the relatively small sample size and its retrospective nature, the results support the effectiveness of combining M-TEER and LAAC in anatomically unselected patients, with a benefit in terms of bleedings at mid-term. Larger, prospective, and adequately powered studies are awaited to confirm these findings.

## 5. Limitation

This was a retrospective, single-center study, and selection bias cannot be excluded. Nevertheless, baseline characteristics were homogenous between the study groups, with the experimental arm showing a higher, predicted bleeding risk. Finally, the relatively small sample size limits the generalization of the results.

## 6. Conclusions

In patients affected by concomitant MR and AF deemed eligible for M-TEER and LAAC, combining M-TEER and LAAC in a single procedure was safe and effective with similar rates of all-cause death, HF rehospitalization, stroke or systemic embolism, and bleedings at 1 year compared to patients undergoing isolated M-TEER. Bleedings were significantly lower in patients receiving LAAC. Larger, prospective studies are required to confirm these findings.

## Figures and Tables

**Table 1:** Baseline characteristics.

	Overall (*n* = 99)	M-TEER+LAAC (*n* = 41)	Isolated M-TEER (*n* = 58)	*p*-Value
rdFemale gender, *n* (%)	45 (45.4)	17 (41.4)	28 (48.2)	0.503
Age, years, median (IQR)	79 (73–82)	78 (72–82)	79 (74–82)	0.582
Hypertension, *n* (%)	83 (83.8)	36 (87.8)	47 (81.0)	0.361
Diabetes mellitus, *n* (%)	32 (32.3)	16 (39.0)	16 (27.5)	0.231
Dyslipidaemia, *n* (%)	53 (53.5)	26 (63.4)	27 (46.5)	0.098
CKD, *n* (%)	61 (61.6)	30 (73.1)	31 (53.4)	0.047
Left ventricular EF (%)	40 (30–55)	45 (31–55)	40 (30–53)	0.796
CHA2DS2-VASc score (points)	4 (3–5)	5 (4–5)	4 (3–5)	0.052
HAS-BLED score (points)	3 (2–4)	3 (3–4)	2.5 (2–4)	<0.004
Previous stroke, *n* (%)	6 (6.1)	1 (2.4)	5 (8.6)	0.402
Previous TIA, *n* (%)	6 (6.1)	3 (7.3)	3 (5.1)	0.708
Previous PCI, *n* (%)	27 (27.2)	14 (34.1)	13 (22.4)	0.197
Previous CABG, *n* (%)	8 (8.1)	5 (12.1)	3 (5.1)	0.207
Coronary artery disease, *n* (%)	38 (38.3)	20 (48.7)	18 (31.0)	0.074
Previous myocardial infarction, *n* (%)	22 (22.2)	10 (24.3)	12 (20.6)	0.663
Degenerative mitral regurgitation, *n* (%)	22 (22.2)	9 (21.9)	13 (22.4)	0.951
Functional mitral regurgitation, *n* (%)	67 (67.6)	28 (68.3)	39 (67.2)	0.913
Mixed mitral regurgitation, *n* (%)	10 (10.1)	4 (9.7)	6 (10.3)	0.922
EuroScore II (%)	6.41 (3.4–8.2)	8 (4.4–8.2)	5.6 (2.8–8.4)	0.081
STS Score (%)	5.6 (3.5–7.8)	7.7 (4–8)	5.3 (3.3–7.4)	0.223
Procedural data				
Amount of contrast, (ml)	50 (30–90)	100 (67–160)	30 (20–50)	<0.001
Fluoroscopy time (min)	22 (14–30)	21 (14–28)	23 (12–32)	0.643
Procedural time (min)	72 (68–75)	75 (63–81)	71 (61–78)	0.242
Mitraclip procedures, *n* (%)	97 (97.9)	39 (95.1)	58 (100)	0.891
Pascal procedures, *n* (%)	2 (2.1)	2 (4.8)	0 (0)	0.893
M-TEER device implanted, *n* (%)	1 (1–2)	1 (1–2)	1 (1–2)	0.083
LoS, days, median (IQR)	5 (3–7)	5 (4–8)	5 (3–6)	0.361
Therapy at discharge				
DAPT, *n* (%)	36 (36.3)	36 (87.8)	0 (0.0)	<0.001
SAPT, *n* (%)	1 (1.1)	1 (2.4)	0 (0.0)	0.234
Anticoagulation, *n* (%)	54 (54.5)	4 (9.7)	50 (86.2)	<0.001
NOAC, *n* (%)	41 (41.4)	3 (7.3)	38 (65.5)	<0.001
VKA, *n* (%)	13 (13.1)	1 (2.4)	12 (20.6)	<0.001
SAPT+NOAC, *n* (%)	8 (8.1)	0 (0.0)	8 (13.7)	0.012

Abbreviations: CKD, chronic kidney disease; PCI, percutaneous coronary intervention; EF, ejection fraction; CABG, coronary artery bypass grafting; DAPT, dual antiplatelet therapy; LoS, length of stay; NOAC, non-vitamin K oral anticoagulants; SAPT, single antiplatelet therapy; TIA, transient ischemic attack; VKA, vitamin K antagonist.

**Table 2:** Procedural characteristics of left atrial appendage closure.

Morphology	
Chicken wing, *n* (%)	22 (53.6)
Cactus, *n* (%)	5 (12.2)
Windsock, *n* (%)	7 (17.1)
Cauliflower, *n* (%)	7 (17.1)
Access sheath	
Double curve, *n* (%)	35 (85.4)
Single curve, *n* (%)	3 (7.3)
Anterior curve, *n* (%)	2 (4.9)
Amulet TorqueVue, *n* (%)	1 (2.4)
Device failure, *n* (%)	1 (2.4)
Device implanted, *n* (%)	40 (97.6)
Amulet 28 mm, *n* (%)	1 (2.5)
Watchman FLX 20 mm, *n* (%)	2 (5.0)
Watchman FLX 24 mm, *n* (%)	10 (25)
Watchman FLX 27 mm, *n* (%)	12 (30.0)
Watchman FLX 31 mm, *n* (%)	11 (27.5)
Watchman FLX 35 mm, *n* (%)	4 (10.0)
Indications to perform LAAC	
Symptomatic hemorrhage, *n* (%)	9 (21.9)
Need for triple antithrombotic therapy, *n* (%)	5 (12.2)
Gastrointestinal bleeding, *n* (%)	5 (12.2)
Oral anticoagulation intolerance, *n* (%)	1 (2.4)
Chronic kidney disease, *n* (%)	30 (73.1)
Stroke/TIA in anticoagulation therapy, *n* (%)	1 (2.4)
TOE findings at 45 days follow-up	
Device related thrombus, *n* (%)	1 (2.5)
Device embolization, *n* (%)	0 (0.0)
Leaks, *n* (%)	0 (0.0)
Pericardial effusion, *n* (%)	0 (0.0)

Abbreviations: LAAC, left atrial appendage closure; TIA, transient ischemic attack; TOE, trans-oesophageal echocardiogram.

**Table 3:** In-hospital outcomes. M-TEER, mitral transcatheter edge-to-edge repair; LAAC, left atrial appendage closure.

	Overall (*n* = 99)	M-TEER+LAAC (*n* = 41)	Isolated M-TEER (*n* = 58)	*p*-Value
In-hospital death	0 (0.0)	0 (0.0)	0 (0.0)	-
Major vascular complications °	1 (1.0)	1 (2.4)	0 (0.0)	0.416
Minor vascular complications	12 (12.1)	7 (17.0)	5 (8.6)	0.201
Local hematoma	9 (9.1)	5 (12.1)	4 (6.9)	0.367
Other than local hematoma ^~^	3 (3.0)	2 (4.9)	1 (1.7)	0.367
Cardiac perforation	0 (0.0)	0 (0.0)	0 (0.0)	-
Device embolization	0 (0.0)	0 (0.0)	0 (0.0)	-
Acute kidney injury	0 (0.0)	0 (0.0)	0 (0.0)	-
Stroke	0 (0.0)	0 (0.0)	0 (0.0)	-

° Surgically treated pseudoaneurysm. ^~^ Three arteriovenous fistulas treated with femoral balloon angioplasty.

**Table 4:** Multivariable logistic regression analyses of baseline characteristics associated with the primary composite endpoint.

	HR (95% CI)	*p*-Value
Male	1.24 (0.48–3.18)	0.642
Diabetes mellitus	1.47 (0.50–4.27)	0.473
Hypertension	0.92 (0.24–3.47)	0.901
CHA2DS2-VASc score	0.75 (0.44–1.29)	0.318
HAS-BLED score	0.88 (0.42–1.81)	0.733
Left ventricular EF	1.00 (0.96–1.03)	0.987
Previous stroke	2.44 (0.30–19.37)	0.392
Coronary artery disease	1.90 (0.69–5.23)	0.211
Baseline NYHA class	1.16 (0.43–3.16)	0.756
CKD	0.85 (0.25–2.87)	0.792
STS Score	1.04 (0.97–1.11)	0.212

Abbreviations: CI, confidence interval; CKD, chronic kidney disease; HR, hazard ratio; EF, ejection fraction; NYHA, New York Heart Association; STS, Society of Thoracic Surgeons.

## Data Availability

Data available based on motivated requests.
